# Evolutionary History of the Global Emergence of the *Escherichia coli* Epidemic Clone ST131

**DOI:** 10.1128/mBio.02162-15

**Published:** 2016-03-22

**Authors:** Nicole Stoesser, Anna E. Sheppard, Louise Pankhurst, Nicola De Maio, Catrin E. Moore, Robert Sebra, Paul Turner, Luke W. Anson, Andrew Kasarskis, Elizabeth M. Batty, Veronica Kos, Daniel J. Wilson, Rattanaphone Phetsouvanh, David Wyllie, Evgeni Sokurenko, Amee R. Manges, Timothy J. Johnson, Lance B. Price, Timothy E. A. Peto, James R. Johnson, Xavier Didelot, A. Sarah Walker, Derrick W. Crook, Modernizing Medical Microbiology Informatics Group (MMMIG)

**Affiliations:** a Modernizing Medical Microbiology Consortium, Nuffield Department of Medicine, John Radcliffe Hospital, University of Oxford, Oxford, United Kingdom; b Cambodia-Oxford Medical Research Unit, Angkor Hospital for Children, Siem Reap, Cambodia; c Icahn Institute and Department of Genetics and Genomic Sciences, Icahn School of Medicine, Mount Sinai, New York, New York, USA; d Wellcome Trust Center for Human Genetics, Oxford, United Kingdom; e Infection Innovative Medicines Unit, AstraZeneca R&D Boston, Waltham, Massachusetts, USA; f Lao-Oxford-Mahosot Hospital Wellcome Trust Research Unit, Mahosot Hospital, Vientiane, Lao People’s Democratic Republic (Laos); g Department of Microbiology, University of Washington, Seattle, Washington, USA; h University of British Columbia, School of Population and Public Health, Vancouver, British Columbia, Canada; i College of Veterinary Medicine, University of Minnesota, St. Paul, Minnesota, USA; j Translational Genomics Research Institute (TGen) North, Flagstaff, Arizona, USA; k Minneapolis Veterans Affairs Health Care System, Minneapolis, Minnesota, USA; l Department of Medicine, University of Minnesota, Minneapolis, Minnesota, USA; m Department of Infectious Disease Epidemiology, School of Public Health, Imperial College London, London, United Kingdom

## Abstract

*Escherichia coli* sequence type 131 (ST131) has emerged globally as the most predominant extraintestinal pathogenic lineage within this clinically important species, and its association with fluoroquinolone and extended-spectrum cephalosporin resistance impacts significantly on treatment. The evolutionary histories of this lineage, and of important antimicrobial resistance elements within it, remain unclearly defined. This study of the largest worldwide collection (*n* = 215) of sequenced ST131 *E. coli* isolates to date demonstrates that the clonal expansion of two previously recognized antimicrobial-resistant clades, C1/*H*30R and C2/*H*30Rx, started around 25 years ago, consistent with the widespread introduction of fluoroquinolones and extended-spectrum cephalosporins in clinical medicine. These two clades appear to have emerged in the United States, with the expansion of the C2/*H*30Rx clade driven by the acquisition of a *bla*_CTX-M-15_-containing IncFII-like plasmid that has subsequently undergone extensive rearrangement. Several other evolutionary processes influencing the trajectory of this drug-resistant lineage are described, including sporadic acquisitions of CTX-M resistance plasmids and chromosomal integration of *bla*_CTX-M_ within subclusters followed by vertical evolution. These processes are also occurring for another family of CTX-M gene variants more recently observed among ST131, the *bla*_CTX-M-14/14-like_ group. The complexity of the evolutionary history of ST131 has important implications for antimicrobial resistance surveillance, epidemiological analysis, and control of emerging clinical lineages of *E. coli*. These data also highlight the global imperative to reduce specific antibiotic selection pressures and demonstrate the important and varied roles played by plasmids and other mobile genetic elements in the perpetuation of antimicrobial resistance within lineages.

## IMPORTANCE

*Escherichia coli*, perennially a major bacterial pathogen, is becoming increasingly difficult to manage due to emerging resistance to all preferred antimicrobials. Resistance is concentrated within specific *E. coli* lineages, such as sequence type 131 (ST131). Clarification of the genetic basis for clonally associated resistance is key to devising intervention strategies. We used high-resolution genomic analysis of a large global collection of ST131 isolates to define the evolutionary history of extended-spectrum beta-lactamase production in ST131. We documented diverse contributory genetic processes, including stable chromosomal integrations of resistance genes, persistence and evolution of mobile resistance elements within sublineages, and sporadic acquisition of different resistance elements. Both global distribution and regional segregation were evident. The diversity of resistance element acquisition and propagation within ST131 indicates a need for control and surveillance strategies that target both bacterial strains and mobile genetic elements.

## INTRODUCTION

Resistance to extended-spectrum cephalosporins in extraintestinal pathogenic *Escherichia coli* (ExPEC) represents a major clinical challenge and is commonly caused by the presence of extended-spectrum beta-lactamases (ESBLs). The majority of ESBL-associated *E. coli* infections are due to a recently emerged, globally distributed ExPEC clone, sequence type 131 (ST131) (1). ST131 predominantly corresponds to serogroup O25b (2, 3) or O16 (4) and belongs to phylogenetic group B2 (5, 6). It remains unclear which features of this clone have resulted in its recent widespread clinical dominance, although antimicrobial resistance and virulence factors are suspected contributors (7).

The *bla*_CTX-M-15_ beta-lactamase gene is the dominant ESBL gene in ST131, but other genetically divergent CTX-M genes also occur in this ST, particularly *bla*_CTX-M-14/14-like_ variants, e.g., in Canada, China, and Spain (8, 9). The almost contemporaneous identification of *bla*_CTX-M_ in ST131 strains from multiple geographic locations suggests repeated acquisition via multiple horizontal gene transfer events (10). Consistently, both *bla*_CTX-M-15_ and *bla*_CTX-M-14/14-like_ variants occur on conjugative plasmids, especially multireplicon IncFII plasmids additionally harboring FIA/FIB replicons (11).

Other data, however, suggest that the widespread distribution of these genes is mediated by clonal expansion of CTX-M-containing strains and global dissemination (12). This is also a plausible hypothesis, since CTX-M plasmids can be inherited stably and *bla*_CTX-M-15_ and *bla*_CTX-M-14_ variants can also integrate into the chromosome (13–15). Nevertheless, clonal expansion of *E. coli* strains with chromosomally integrated *bla*_CTX-M_ has not yet been demonstrated.

Two recent studies used whole-genome sequence (WGS) data to investigate the population structure of ST131. The first found that ST131 expansion in the United States has been driven by a single sublineage, *H*30, defined by the presence of a specific fimbrial adhesin allele, *fimH30*. Within *H*30, nested clades have emerged: *H*30R, containing mutations in the chromosomal genes *gyrA* and *parC* that confer fluoroquinolone resistance, and *H*30Rx, containing the same *gyrA* and *parC* mutations but additionally associated with *bla*_CTX-M-15_ (13). The second study (16), which included samples from six locations around the world, resolved the ST131 population structure into three clades, A, B, and C, with clade C comprising two subgroups, C1 and C2, corresponding to the *H*30R and *H*30Rx clades. However, this study included only four isolates from Asia, where ESBL ExPEC prevalence may be highest (17, 18). Furthermore, neither study directly tested the competing hypotheses that ESBL dissemination in ST131 has occurred through multiple horizontal gene transfer events versus clonal expansion.

Here, we used a broader set of ST131 WGS data, including many more isolates from Asia and *bla*_CTX-M-14/14-like_-containing strains, alongside a subset of CTX-M plasmid sequences, to estimate the contribution of each potential route of dissemination to the worldwide prevalence of ST131.

## RESULTS

The 215 ST131 genome sequences analyzed included 67 strains from various locations in Southeast Asia, 33 from Oxford in the United Kingdom, 11 from a global resistance surveillance program at AstraZeneca, 8 from Canada, and 96 predominantly North American isolates previously reported by Price et al. (13) (details on new isolates are shown in Table S1 in the supplemental material; these strains included both human and animal isolates and clinical and carriage isolates.)

### Asian ST131 strains are consistent with the previously described core phylogeny, and the C1/*H*30 and C2/*H*30Rx clades emerged from a North American ancestor.

For the 4,717,338 sites in the SE15 ST131 reference genome (19), the mean mapping call rate across the data set was 93.3%. In total, 40,057 (0.85%) sites were variable, with 6,879 (0.15%) representing core single-nucleotide variants (SNVs) called in all 215 isolates. Overall, 611,770 (13%) sites were in recombinant regions, including 4,120 core SNVs, leaving 2,759 core nonrecombinant SNVs for phylogenetic analysis.

**FIG 1:**
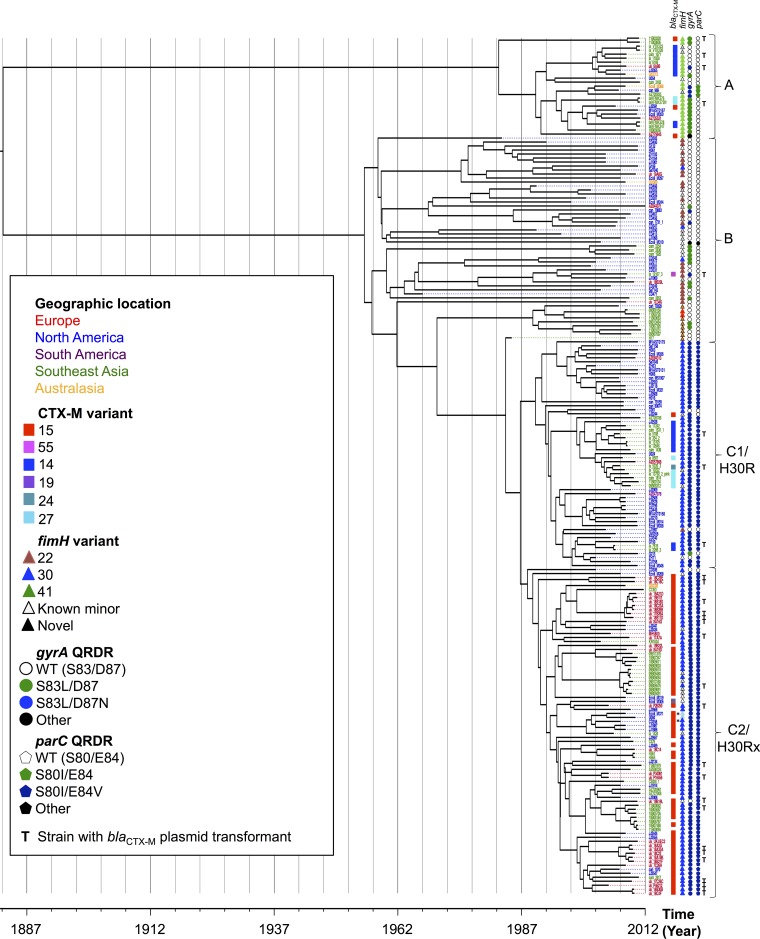
Time-scaled phylogeny of ST131 *E. coli* (*n* = 215), with associated *bla*_CTX-M_/*fimH* variants, and quinolone resistance-determining region (QRDR) mutations in *gyrA* (WT, wild-type QRDR). Curly brackets represent ST131 clades as described in the text. Tips are colored by geographic region, per the key. **T**, *bla*_CTX-M_ plasmid transformant generated for strain; *, cases with putative deletions in the assembled *bla*_CTX-M-15_ gene.

Consistent with the two previous WGS-based ST131 phylogenies (13, 16), the time-scaled phylogeny inferred from this ST131 data set (which included >10 times more Asian isolates than considered previously) comprised three clades (Fig. 1), A (*n* = 25), B (*n* = 51), and C (*n* = 139), with C containing two subclades, C1 (*n* = 57) and C2 (*n* = 82), characterized by the presence versus absence, respectively, of *bla*_CTX-M-15_ (16). Isolates from all geographic regions were identified within each clade, although there were smaller, geographically restricted clusters within these (Fig. 1, tip color). This supports both global transmission and localized lineage expansion following specific introductions into a geographic locality.

The estimated time to most recent common ancestor (TMRCA) for the whole genomic data set was ~130 years ago, when clade A diverged from clades B and C. Twenty-five years ago, clade C emerged out of the paraphyletic clade B, which was quickly followed by the split between subclades C1 and C2. The number of core SNVs separating the clades was approximately 250 for clade A versus clades B and C, 50 to 60 for clade B versus clades C1 and C2, and 10 to 30 for clade C1 versus clade C2. The evolutionary rate of ST131 was estimated in BEAST (see Materials and Methods) at 2.46 × 10^−7^ mutations per site per year (95% confidence interval [CI], 2.18 × 10^−7^ to 2.75 × 10^−7^), equating to 1.00 (95% CI, 0.89 to 1.12) mutation per genome per year.

All possible geographic origins of the root of the ST131 lineage were inferred to be equally likely since the root is far back in time relative to the estimated migration rates. Clade A was inferred to originate in Southeast Asia with ~70% confidence (78% when the unsampled deme was included in the model [see Materials and Methods]), and the B/C clades were inferred to originate from North America with ~88% confidence. The ancestral origin of C1/*H*30 and C2/*H*30Rx was strongly inferred as being in North America (98% confidence; 85% confidence when the unsampled deme was included in the model) with subsequent dissemination to Europe and Asia. Locations of more recent nodes are inferred with high confidence, as expected (20).

### *bla*_CTX-M_, *fimH*, and *gyrA* variants are strongly associated with specific ST131 clades.

Overall, 105 (49%) ST131 isolates harbored *bla*_CTX-M_, including 74 isolates (34%) with *bla*_CTX-M-15_, 20 (9%) with *bla*_CTX-M-14_, 8 (4%) with *bla*_CTX-M-27_, and one each (0.4%) with *bla*_CTX-M-19_, *bla*_CTX-M-24_, and *bla*_CTX-M-55_ (Fig. 1). *bla*_CTX-M-15_ was almost completely restricted to the C2 clade, as described previously (16), occurring in 69/82 (84%) C2 isolates but only sporadically in other clades (4/133; *P* < 0.001, Fisher exact test). *bla*_CTX-M-14_ and *bla*_CTX-M-27_ were also clustered within the two different clades A and C1 and absent from B and C2 (Fig. 1). Overall, the presence of shared *bla*_CTX-M_ variants within clusters was constrained to those with a TMRCA of less than 25 years, suggestive of the emergence of *bla*_CTX-M_ within ST131 after the widespread introduction of extended-spectrum cephalosporins in clinical practice.

The most common *fimH* variant was *fimH30* (*n* = 123; 57%), followed by *fimH22* (*n* = 24; 11%) and *fimH41* (*n* = 21; 10%), whereas 23 isolates had novel *fimH* variants, and one was *fimH* null. As observed for *bla*_CTX-M_, *fimH* alleles were strongly associated with clade, with 21/25 (84%) isolates in clade A having *fimH41*, 23/51 (45%) in clade B having *fimH22*, and 122/139 (88%) in clade C having *fimH30* (*P* < 0.001; Fisher exact test).

Fluoroquinolone resistance mutations in *gyrA* and *parC* were also clade associated, with isolates in clades A and B typically having no or only single mutations in these genes’ quinolone resistance-determining regions (QRDRs) (Fig. 1). In contrast, most clade C isolates had double mutations in both *gyrA* and *parC*, shown to confer high-level fluoroquinolone resistance (21) (132/139 [95%]). The seven clade C isolates without these mutations (5 in C1 and 2 in C2) were sporadic, with two having non-*fimH30* variants, suggesting intermittent recombination events affecting *gyrA*, *parC*, and *fimH*. The emergence of this double-mutation, high-level fluoroquinolone resistance genotype was dated by our methods to 25 to 40 years ago, consistent with the introduction of fluoroquinolones in clinical practice.

### *bla*_CTX-M-15_ in clade C2 is present in a consistent but short flanking structure, frequently truncated by IS*26* elements and within different genetic backgrounds.

In four of the 74 *bla*_CTX-M-15_-containing isolates, *bla*_CTX-M-15_ was present on two different contigs (C1353, JJ2643, CD358, and JJ2434). In another isolate (JJ2547), the assembled contig with *bla*_CTX-M-15_ contained a series of N’s, suggesting possible uncertainty around the contig assembly or multiple locations of the gene. These five isolates were excluded from further analysis of flanking regions. In the 69 remaining isolates (3 in clade A, 1 in clade C1, and 65 in clade C2), *bla*_CTX-M-15_ was found downstream of a homologous tract of 48 bp preceded by an IS*Ecp1* right-end inverted repeat region (IRR-R) and upstream of a homologous tract of 46 bp followed by ORF477. This is consistent with the introduction of an IS*Ecp1*-*bla*_CTX-M-15_-ORF477 unit within ST131 and subsequent rearrangement events affecting this structure.

**FIG 2:**
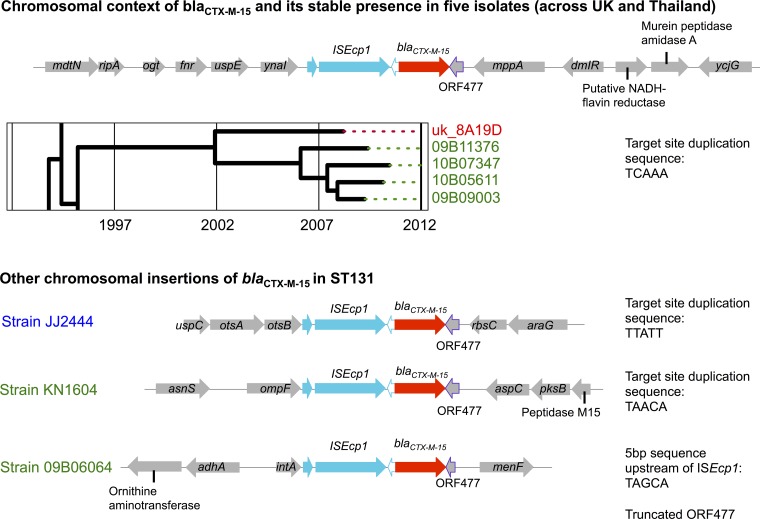
Chromosomal location of IS*Ecp1*-mediated *bla*_CTX-M-15_ insertion events and evidence of acquisition and evolution by descent (inset phylogeny) over approximately 8 years across two geographic regions (Oxford, United Kingdom; Mae Sot, Thailand-Myanmar border). Coloring of isolate names represents geographic location (red, Europe; green, Southeast Asia; blue, North America).

In clade C2, *bla*_CTX-M-15_ was integrated into the chromosome of 8/65 (12%) isolates, with four unique integration events, one of which was stably present in a subcluster of five isolates with a common ancestor around 2002 and spread across two geographical regions (Fig. 2). All chromosomal integration events were associated with an intact IS*Ecp1* upstream of *bla*_CTX-M-15_. In three isolates, the IS*Ecp1*-*bla*_CTX-M-15_-ORF477 unit was flanked by 5-bp target site duplications consistent with transposition, and in one isolate, the ORF477 was truncated, suggestive of either one-ended transposition (22) or standard transposition followed by a deletion event (Fig. 2).

**FIG 3:**
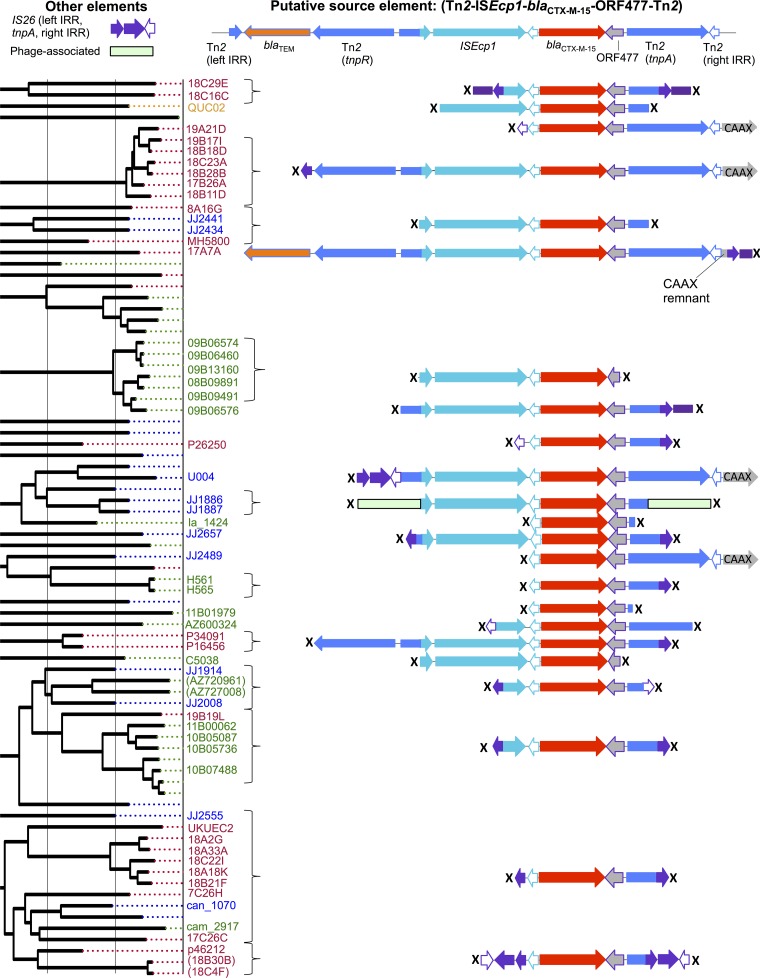
Genetic flanking context of *bla*_CTX-M-15_ region for clade C2/*H*30Rx isolates. (Top) Putative source element. (Left) C2/*H*30Rx phylogeny. Many contexts are limited by the extent of the assembled region around the *bla*_CTX-M-15_ gene (marked with “X”). For all similarly colored, vertically aligned regions below the “Putative source element,” sequence identity is 100%. Curly brackets cluster those isolates with identical flanking sequences. Flanking contexts are not shown and tip labels are omitted for isolates with known chromosomal integration of *bla*_CTX-M_, for *bla*_CTX-M_-negative isolates in the clade, or for isolates where the flanking sequence was not evaluable (see Results).

In 27 of the 57 remaining C2 isolates, *bla*_CTX-M-15_ appeared plasmid associated, being either present in plasmid transformants (*n* = 20) or flanked by likely plasmid-associated sequences in the contig assemblies (*n* = 7). In the remaining 30 isolates, the location of *bla*_CTX-M-15_ could not be defined due to limitations of the short-read assemblies. In all 57 isolates, the upstream sequence was either an intact or truncated IS*Ecp1* sequence, and in 51/57 (89%) isolates, the sequence downstream of ORF477 was either an intact or a truncated Tn*2* structure (Fig. 3). In 12 isolates distributed throughout clade C2, a continuation of the Tn*2* sequence was also observed upstream of the IS*Ecp1* sequence, consistent with the IS*Ecp1* element (flanked by a pair of 5-bp repeats, all TCATA) being nested within a complete or partial Tn*2* transposon. In 40/57 (65%) isolates, IS*26* repeat regions truncated either or both of these upstream and downstream contexts (Fig. 3).

### *bla*_CTX-M-14_ and *bla*_CTX-M-27_ are present in diverse genetic backgrounds and within a common IS*Ecp1*-IS*903B* transposition unit.

For *bla*_CTX-M-14_, evidence of chromosomal integration was also found: two related *bla*_CTX-M-14_ isolates had IS*Ecp1*-mediated chromosomal integration of *bla*_CTX-M-14_ downstream of the *gatY* gene (clade A, isolates HFMK328 and HFMK347). Six isolates had plasmid-associated *bla*_CTX-M-14_ based on annotated flanking sequences/transformants, whereas for the rest (*n* = 14), the location of *bla*_CTX-M-14_ was uncertain due to limitations of the *de novo* assemblies.

In all these isolates, IS*Ecp1* was consistently located upstream of *bla*_CTX-M-14_, as with *bla*_CTX-M-15_, but at only a 43-bp distance, and the downstream flanking sequences were composed of either intact or truncated IS*903B* elements. In clade A, the genetic flanking sequences surrounding *bla*_CTX-M-14_ were consistent with the host strain subcluster and were homologous over the observed contig length within this subcluster (see Fig. S1 in the supplemental material, clade A CTX-M-14 subcluster [i]). This supports a single *bla*_CTX-M-14_ plasmid acquisition event followed by either evolution with plasmid inheritance or subsequent transfer of a *bla*_CTX-M-14_-containing genetic unit within the subcluster. The flanking sequence for isolate la_5108_T in clade C1 also incorporated an IS*Ec23* element downstream of IS*903B* and was homologous to that in clade A CTX-M-14 subcluster [i] (see Fig. S2), suggesting horizontal transfer of this genetic unit between clades.

Six of eight isolates with *bla*_CTX-M-27_ were closely related in clade C1, again supporting a single plasmid acquisition event. However, they also all contained bilateral truncation of the IS*Ecp1*-*bla*_CTX-M_-IS*903B* structure by IS*26* elements, which occurred in four different contexts, suggesting frequent IS*26*-mediated *bla*_CTX-M-27_ transposition events within this subcluster (see Fig. S2 in the supplemental material).

### Plasmid replicon analysis demonstrates a degree of clade-associated plasmid segregation suggestive of ancient IncF plasmid acquisition events.

The predominant replicon family was IncF, identified in 206/215 ST131 isolates (96%). Specific IncF variants differed in frequency, with FII found in 199/215 isolates (93%), FIB in 155/215 (72%), FIA in 145/215 (67%), and FIC in 17/215 (8%). Specific IncF replicons and combinations thereof were clade associated (Table 1). A number of non-F Inc types were also identified; of these, IncH was associated with clade B and IncI was associated with clade C1 (Table 1). Col-like plasmids were also common (189/215 isolates [88%]); however, there was no clear association of any Col type with clade (see Fig. S3 in the supplemental material).

**TABLE 1:** Plasmid replicon families/types by clade

Inc type	No. of isolates (row %) for clade:				Total no. of isolates	Difference in replicon prevalence across clades, *P*
	A/*H*41	B/*H*22	C1/*H*30R	C2/*H*30Rx		
A/C		1 (33)	1 (33)	1 (33)	3	1
B/O/K/Z	1 (10)	2 (20)	2 (20)	5 (50)	10	0.9
FIA only			1 (50)	1 (50)	2	1
FIA total	15 (10)	1 (0.7)	52 (36)	77 (53)	145	**<0.001**^a^
FIA-FIB			3 (100)		3	0.07
FIA-FII			4 (9)	39 (91)	43	**<0.001**
FIA-FIB-FII	15 (15)	1 (1)	44 (45)	37 (38)	97	**<0.001**
FIB only		2 (100)			2	0.12
FIB total	25 (16)	43 (28)	47 (30)	40 (25)	155	**<0.001**
FIB-FII	8 (22)	25 (69)		3 (8)	36	**<0.001**
FIB-FIC-FII	2 (12)	15 (88)			17	**<0.001**
FIC total	2 (12)	15 (88)			17	**<0.001**
FII only		4 (67)	1 (17)	1 (17)	6	0.12
FII total	25 (13)	45 (23)	49 (25)	80 (40)	199	**0.02**
H		5 (100)			5	**0.001**
I	4 (25)	6 (38)	3 (19)	3 (19)	16	0.09
N		2 (15)	7 (54)	4 (31)	13	0.15
P		2 (100)			2	0.12
Q	1 (11)		7 (78)	1 (11)	9	**0.005**
R		1 (100)			1	0.36
X-like		7 (58)	2 (17)	3 (25)	12	0.06
Y	1 (8)	1 (8)	2 (17)	8 (67)	12	0.25

a Boldface indicates statistically significant differences (*P* < 0.05).

A specific FII variant (GenBank nucleotide sequence accession no. AY458016; pC15-1a; consistent with pMLST allele 2) was significantly associated with clade C2 (48/82 C2 isolates versus 18/153 non-C2 isolates, *P* < 0.001, Fisher exact test). Within clade C2, a further 23 isolates had eight different FII_AY458016-like variants containing up to 12 SNVs among them; almost all of these variants were in isolates with FIA-FIB-FII replicon combinations (see Fig. S3 in the supplemental material). Of the 11 clade C2 isolates without FII_AY458016-like replicon variants, four contained a plasmid with a different FII replicon (GenBank nucleotide sequence accession no. AJ851089; pRSB107, 35 SNVs different from FII_AY458016; consistent with pMLST allele 1), five had chromosomally integrated *bla*_CTX-M-15_ (of which four also contained an FII_ AJ851089-like plasmid), one was *bla*_CTX-M_ negative, and one contained deletions in *bla*_CTX-M-15_. There were only nine clade C2 isolates with FII_AY458016-like replicons but no *bla*_CTX-M-15_. The different FII replicon associated with *bla*_CTX-M-15_ in clade C2, FII_AJ851089, was also clade associated, being found predominantly in clades A (13/25 isolates, 52%) and C1 (41/57, 72%) rather than B (12/51, 24%) and C2 (8/82, 10%) (*P* < 0.0001, Fisher exact test) (see Fig. S3). Overall, this strongly suggests the ancestral acquisition of the FII_AY458016 replicon within clade C2, its association with *bla*_CTX-M-15_ and the expansion of the clade, its evolution in the presence of FIA-FIB replicons, and its sporadic loss.

### Plasmid transformants demonstrate similarities and differences in *bla*_CTX-M-15_ plasmids from ST131 clades and other sequence types.

**FIG 4:**
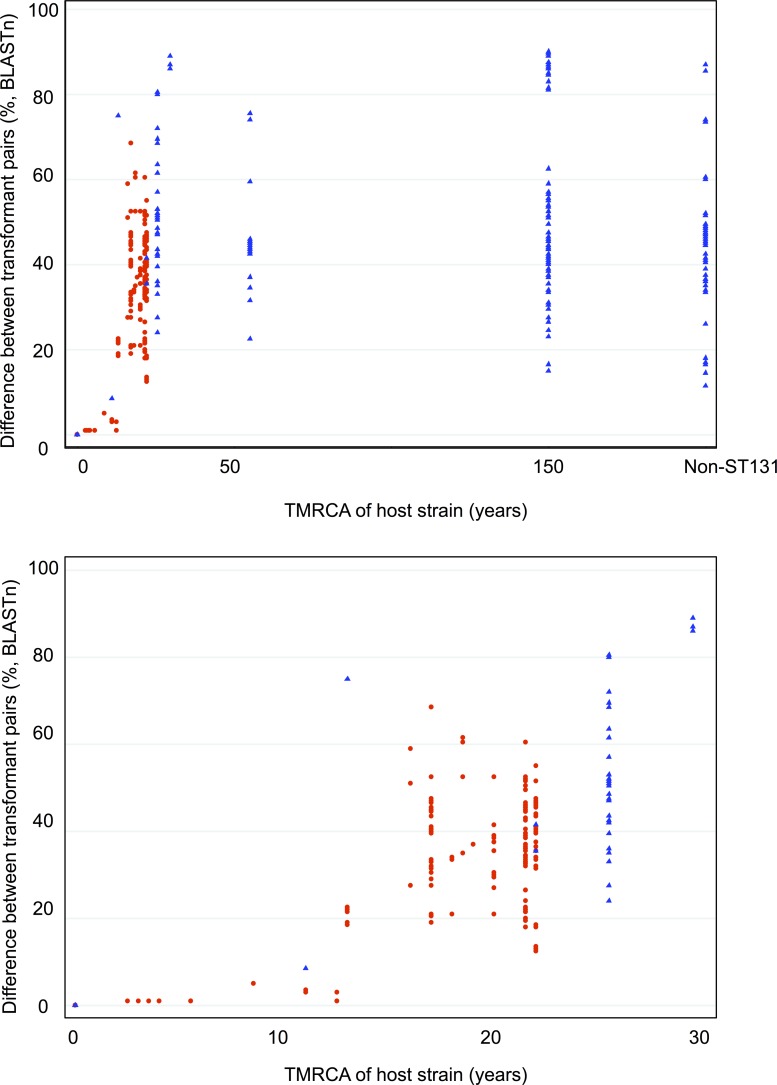
Mean pairwise percent difference between all transformed plasmid sequence pairs plotted against time to most recent common ancestor (TMRCA) for the two strains hosting the respective transformant plasmids. Red circles indicate pairs where both strains are in C2; blue triangles indicate pairs where one or both strains are outside C2. The lower panel represents the same data but limited to strains with a TMRCA of less than 30 years.

Sequence data were generated for 30 transformed *bla*_CTX-M_ plasmids (denoted as <host isolate name> _T; relevant source strains are labeled “T” in Fig. 1): four from clade A, containing *bla*_CTX-M-15_ (*n* = 1), *bla*_CTX-M-14_ (*n* = 2), and *bla*_CTX-M-27_ (*n* = 1); one from clade B, containing *bla*_CTX-M-55_; three from clade C1, containing *bla*_CTX-M-14_ (*n* = 2) and *bla*_CTX-M-24_ (*n* = 1); 20 from clade C2, containing *bla*_CTX-M-15_; and two *bla*_CTX-M-15_ plasmids from non-ST131 isolates (see Table S2 in the supplemental material). The mean percent pairwise differences in plasmid sequence between all plasmid pairs were compared with the divergence times of the corresponding host strains. This demonstrated that all transformed *bla*_CTX-M_ plasmids shared at least 10% homology but could be genetically divergent (Fig. 4), plasmids found in different STs could be very similar (up to ~90% sequence homology), and plasmid genetic similarity correlated with host strain divergence time for recently diverged host strains (up to ~30 years) but was much more variable for more remotely diverged host strains.

**FIG 5:**
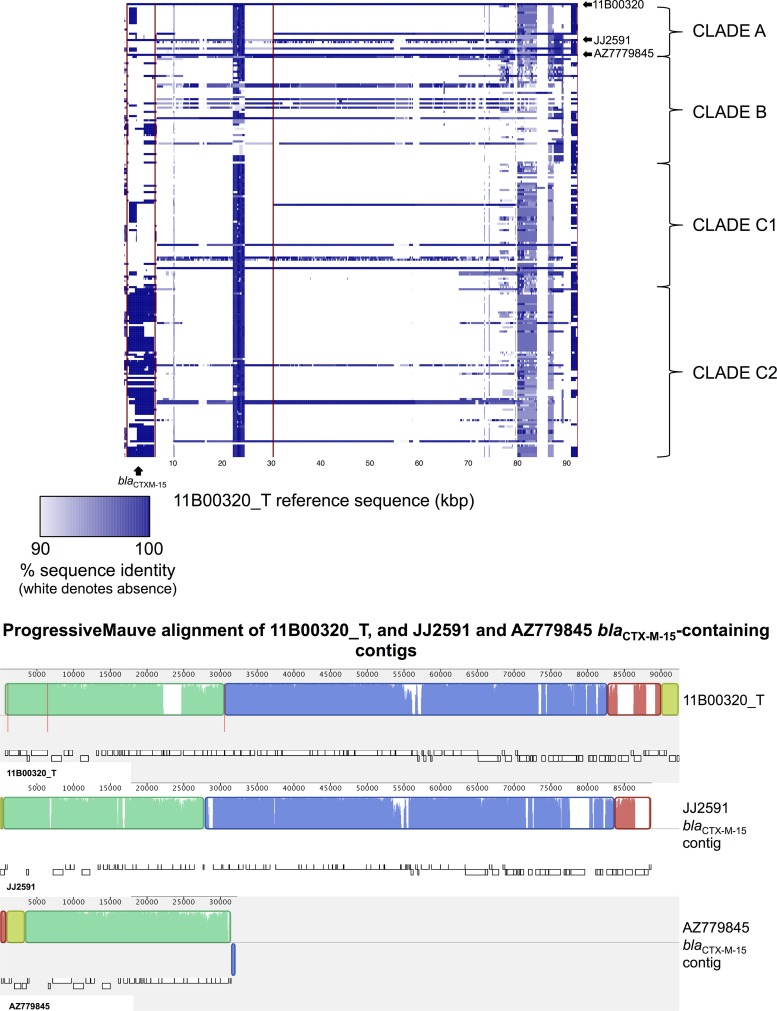
(Top) BLASTn-based comparison across the ST131 data set, using the *bla*_CTX-M-15_-containing 11B00320_T as a reference. Color represents degree of presence/absence of identity to the 11B00320_T sequence on an isolate-by-isolate basis per row. Rows/isolates are arranged as in the Fig. 1 phylogeny. (Bottom) ProgressiveMauve alignment of 11B00320_T and the CTX-M-15-containing contigs for two other isolates in clade A. Alignments with substantial homology are represented as similarly colored blocks (“locally collinear blocks”); white regions within these blocks represent low homology. Vertical red lines represent contig breaks.

Most transformed *bla*_CTX-M_ plasmids were IncF, except in two cases (11B00320_T and la_7619_T). BLASTn-based comparisons revealed that the clade A *bla*_CTX-M-15_ IncI plasmid (11B00320_T; isolated in Mae Sot, Thailand-Myanmar border) was circulating in a limited fashion (Fig. 5) but with substantial sequence homology to *bla*_CTX-M-15_-containing contigs from the two other clade A *bla*_CTX-M-15_-positive isolates (JJ2591, Minneapolis, MN, USA, and AZ779845, Spain). Although we did not have transformants or specific plasmid sequences for these, the *bla*_CTX-M-15_-containing contig assembled for JJ2591 was 88,693 bp long and very similar to the 11B00320_T assembly, whereas the AZ779845 *bla*_CTX-M-15_-containing contig was 32,228 bp long and likewise highly similar in structure (Fig. 5). These data suggest that an IncI-CTX-M-15 plasmid is responsible for sporadic, horizontal introductions of *bla*_CTX-M-15_ into ST131 with a wide geographic distribution.

**FIG 6:**
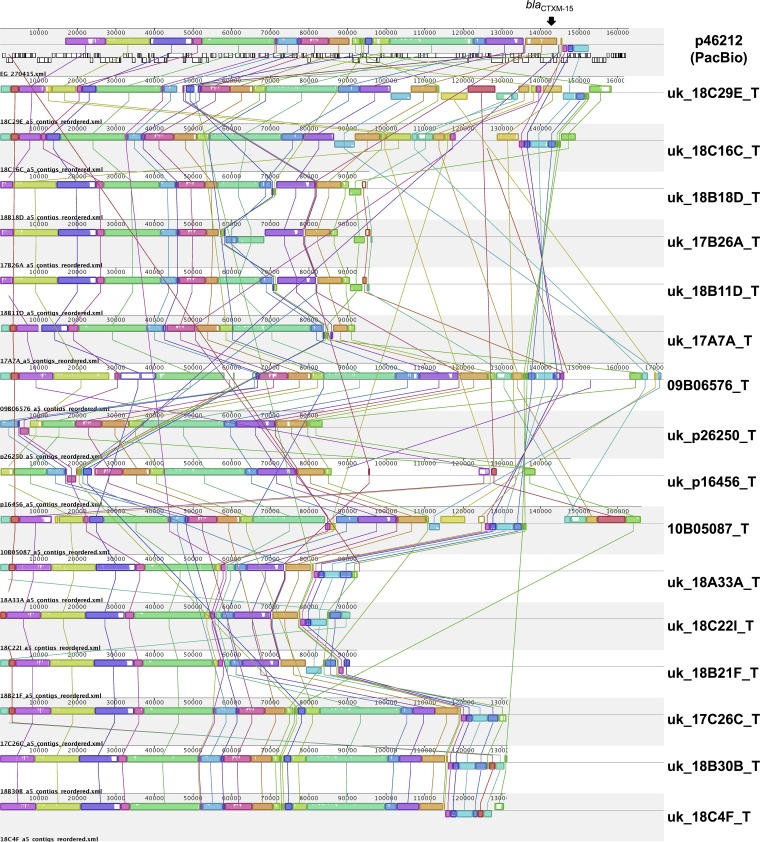
ProgressiveMauve alignment of assembled contigs, ordered with respect to pP46212, for 17 *bla*_CTX-M-15_ FII plasmids derived from sequenced transformants, all in clade C2/*H*30Rx. Plasmids are ordered with respect to the position of their host strains in the main phylogeny (except pP46212 [Fig. 1]). Alignments with substantial homology are represented as colored blocks (“locally collinear blocks”) and are linked with colored lines; white regions within these blocks represent low homology.

Nineteen of 20 transformed *bla*_CTX-M-15_ plasmids from clade C2 contained an FII_ AY458016-like replicon, supporting the association of IncFII_AY458016 with *bla*_CTX-M-15_. Sequence comparisons among 17 (of 20 total) plasmids from clade C2 that contained IncFII_AY458016 identified a significant degree of homology (Fig. 6) (excluding 8A16G_T, 11B01979_T, and 19B19L_T; see Materials and Methods). However, only eight coding sequences were shared with 100% nucleotide similarity, including *bla*_CTX-M-15_, *bla*_OXA-1_, *aac(6')-Ib-cr*, a glucose-1-phosphatase-like-enzyme, a CAAX amino-terminal protease self-immunity protein, a hypothetical phage protein, and a *pemI*/*pemK* plasmid addiction system. This lack of gene conservation suggests that significant genetic exchange and rearrangement occur among these plasmids as they evolve within the subclade.

**FIG 7:**
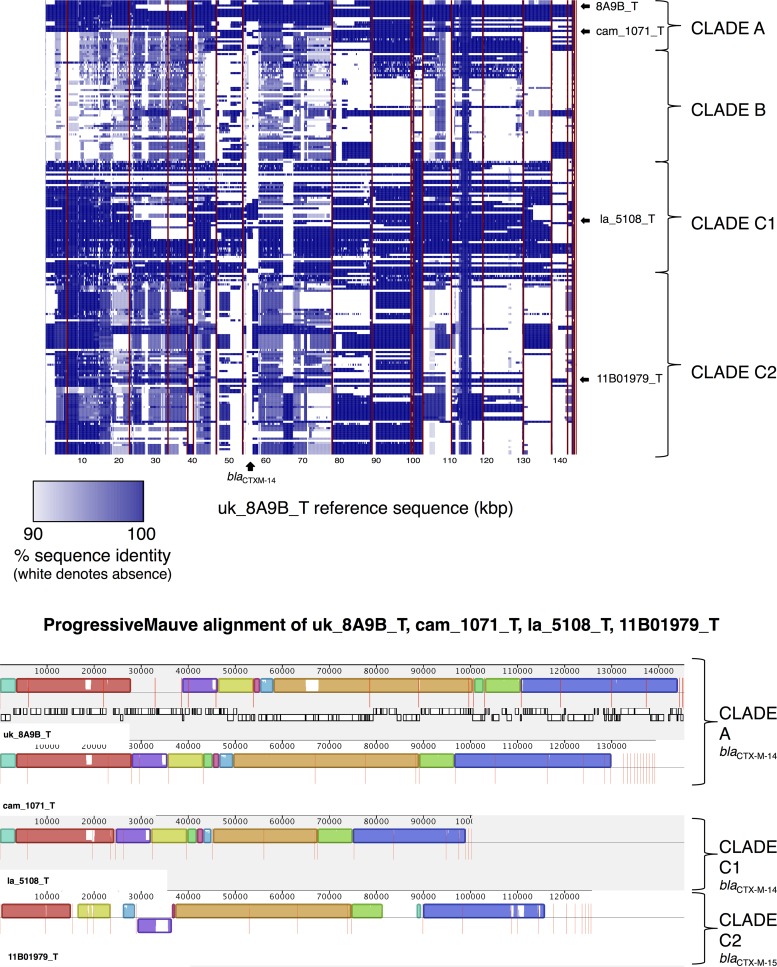
(Top) BLASTn-based comparisons across the ST131 data set, using the *bla*_CTX-M-14_-containing uk_8A9B_T as a reference. Color represents degree of presence/absence of identity to the uk_8A9B_T sequence on an isolate-by-isolate basis per row. Rows/isolates are arranged as in the Fig. 1 phylogeny. (Bottom) ProgressiveMauve alignment of uk_8A9B_T, cam1071_T, la_5108_T, and 11B01979_T, with the last three ordered using uk_8A9B_T as a reference and contig boundaries represented as vertical red lines. Alignments with substantial homology are represented as colored blocks (“locally collinear blocks”); white regions within these blocks represent low homology. Vertical red lines represent contig breaks.

Genetic comparisons among the transformed *bla*_CTX-M-14/14-like_ plasmids revealed that three shared strikingly similar genetic structures, two of which (uk_8A9B_T, Oxford, United Kingdom, and cam_1071_T, Siem Reap, Cambodia) were identified in clade A, in host strains with a TMRCA within the last 15 years, and one in clade C1 (la_5108_T, Vientiane, Laos) (Fig. 7). BLASTn-based comparisons across all 215 ST131 sequences demonstrated that many isolates in clades A (predominantly subcluster [i]) and C1 apparently contained stretches of highly similar genetic structures, as did small numbers of isolates in clade C2 (Fig. 7). 11B01979_T (Mae Sot, Thailand-Myanmar border), a transformed *bla*_CTX-M-15_ plasmid in clade C2, also showed significant homology to uk_8A9B_T, cam_1071_T, and la_5108_T (Fig. 7), suggesting that both *bla*_CTX-M-14_ and *bla*_CTX-M-15_ variants can be accommodated on the same plasmid background.

The isolates containing *bla*_CTX-M-55_ and *bla*_CTX-M-24_ (one-SNV derivatives of *bla*_CTX-M-15_ and *bla*_CTX-M-14_, respectively) apparently resulted from discrete plasmid acquisition and/or *bla*_CTX-M_ transposition events within ST131 (see Fig. S4 in the supplemental material). These were not therefore shown to represent *bla*_CTX-M_ evolution within established *bla*_CTX-M-15_ or *bla*_CTX-M-14_ plasmid backgrounds.

## DISCUSSION

Our WGS analysis of the largest (*n* = 215) and most diverse collection of ST131 isolates to date establishes that the global emergence of drug-resistant clades (C1/*H*30, C2/*H*30Rx) occurred approximately 25 years ago, most likely in a North American context and consistent with strong selection pressure exerted by the widespread introduction and use of fluoroquinolones and extended-spectrum cephalosporins. Interestingly, this appears to be at odds with the previous observation that ESBLs predominating in North America in the 1990s and early 2000s were mostly *bla*_TEM_ or *bla*_SHV_ variants (23); however, the studies summarized in this review were mostly undertaken in nosocomial and/or critical care settings, investigated non-*E. coli Enterobacteriaceae*, used phenotypic screening methods that may have missed the emergence of *bla*_CTX-M_ in *E. coli* (e.g., higher extended-spectrum cephalosporin breakpoints, focused on beta-lactam/beta-lactamase inhibitor-resistant isolates), or did not test specifically for *bla*_CTX-M_ variants.

Although members of each ST131 clade have dispersed globally, our data indicate that within specific geographic regions, smaller clonal ST131 outbreaks occur at all genetic levels (gene, flanking context, plasmid, and host strain), supporting the hypothesis that both horizontal gene transfer and clonal expansion have contributed to the global dissemination of this sequence type. The estimated molecular evolutionary rate of ST131 (1.00 mutation per genome per year) is similar to previous estimates from ST131 (24) and the species overall (25), strongly suggesting that ST131’s epidemiological success is not due to a higher-than-average mutation rate.

Our study shows that the apparent persistence of particular *bla*_CTX-M_ variants within specific ST131 clades is due to diverse mechanisms. These include (i) acquisition of a *bla*_CTX-M_-containing plasmid by a specific host strain subcluster, followed by evolution and spread across geographic regions (e.g., clade A *bla*_CTX-M-14_ subcluster [i] [Fig. 6; see also Fig. S2 in the supplemental material]); (ii) multiple discrete acquisition events involving *bla*_CTX-M_-containing plasmids (e.g., *bla*_CTX-M-55_ and *bla*_CTX-M-24_ [see Fig. S4]; different *bla*_CTX-M-14_ clusters); (iii) horizontal transfer of common plasmid structures across clades (e.g., the IncI *bla*_CTX-M-15_ plasmid [Fig. 5]); and (iv) chromosomal integration of *bla*_CTX-M_ and evolution by descent (e.g., *bla*_CTX-M-15_ [Fig. 2]; *bla*_CTX-M-14_). Despite this high degree of genetic plasticity, we also found clear structuring of *bla*_CTX-M_ variants and plasmid content, with the near-complete absence of *bla*_CTX-M_ in clade B and associations of *bla*_CTX-M-14/14-like_ variants with clade A and clade C1/*H*30R, of *bla*_CTX-M-15_ with clade C2/*H*30Rx, and of specific combinations of IncF replicons with certain clades. This supports the hypothesis that some plasmid replicons are acquired and persist stably within clades. Although the evolutionary dynamics of plasmid-host combinations remain to be clearly elucidated, coevolution of host and plasmid in the case of C2/*H*30Rx appears to have ameliorated costs to the host and facilitated persistence of the replicon (26, 27), with ongoing conjugative exchange of genetic material. The relative contribution of changing environmental influences on this coevolution is unclear; it may also be affected by a host-plasmid “arms race” in a microevolutionary version of the “Red Queen Hypothesis” (antagonistic coevolution) (28, 29).

The almost ubiquitous presence of *bla*_CTX-M-15_ in clade C2/*H*30Rx is most striking and is strongly associated with the presence of an IncFII_AY458016-like replicon. Previous smaller studies have found that *bla*_CTX-M-15_ is frequently part of a 2,971-bp IS*Ecp1*-*bla*_CTX-M-15_-ORF477 transposition unit, with *bla*_CTX-M-15_ located 48 bp downstream of the IS*Ecp1* IRR-R, and that this is commonly nested within a Tn*2* element (30). One hypothesis is that an IncFII_AY458016 ancestral plasmid was acquired by a fluoroquinolone-resistant C1 host strain approximately 25 years ago and subsequently incorporated one of these *bla*_CTX-M-15_ transposition units. In response to the widespread clinical use of extended-spectrum cephalosporins and fluoroquinolones, the C2/*H*30Rx clade has expanded, and within it, *bla*_CTX-M-15_ has been mobilized through further transposition events (e.g., to the chromosome) and rearrangement/recombination among IncFII-like plasmids, much of this associated with IS*26* (31) (Fig. 3). The persistence of the IncFII_AY458016-like replicon in C2 may be attributable, at least in part, to its association with a plasmid addiction system (*pemI/pemK*) (32), whereas its ongoing evolution is potentially linked to the concomitant presence of FIA/FIB replicons on *bla*_CTX-M-15_ plasmids (see Fig. S3 in the supplemental material) (33). Alternative hypotheses could be envisioned, e.g., multiple, clade C2-restricted acquisitions of different *bla*_CTX-M-15_-containing FII_AY458016-like plasmids or recurrent IS*Ecp1*-*bla*_CTX-M-15_-ORF477 unit acquisitions. These seem less likely, however, because (i) there are no geographic or major genotypic distinctions between clades C1 and C2 to explain why this would occur, (ii) there is a degree of homology in the flanking contexts around the gene throughout the clade, and (iii) flanking context/transformed plasmid structures also appear to be consistent within C2 subclusters.

Our novel comparison of transformed, sequenced plasmids demonstrates that a substantial degree of similarity can exist among *bla*_CTX-M_ plasmids found in different clades and STs. This indicates that between-clade/ST transfer of these resistance plasmids occurs and that care is needed when inferring plasmid evolution by descent (Fig. 4). The observed plasmid similarity across geography in the context of host strain phylogenetic clustering and homology in regions flanking *bla*_CTX-M_ (as demonstrated here) is much more likely to represent ancestral plasmid acquisition and subsequent evolution by descent rather than multiple acquisition events but still needs to be interpreted with caution, as it may, for example, represent exposure to a common, global, plasmid reservoir.

The study has several limitations. First is the inability with short-read sequencing and limited transformant sequencing to assess fully the flanking regions and plasmid structures across the entire data set. In particular, the BLASTn-based heat maps across the wider data set represent not genetic contiguity of plasmid structures within isolates as such but instead overall plasmid sequence presence/absence. Second, results from *de novo* assemblies of these short-read data also must be interpreted cautiously, as these assembly methods are known to increase the number of SNVs compared with mapping-based approaches and may result in misinterpretations of genetic structures, particularly repetitive regions (34). Third, again relating to the limitations of short-read data, the transformant plasmid sequences comprise multiple contigs, precluding certainty as to the plasmids’ exact structure. More extensive future use of long-read sequencing (e.g., PacBio) could help resolve this. Fourth, many of our *H*30Rx/C2 clade transformed CTX-M plasmids were from a single United Kingdom center; however, the genetic flanking contexts identified here have also been found in plasmid sequences from other national and international locations (30, 35, 37), suggesting that these are dispersed more widely and that our results are likely generalizable.

In summary, our analysis strongly suggests that the emergence of the C2/*H*30Rx clade within ST131 has been driven by the acquisition of a specific FII plasmid, which has subsequently undergone major genetic restructuring within its globally dispersing bacterial host. The initial acquisition event occurred approximately 25 years ago, possibly associated with the widespread clinical introduction of extended-spectrum cephalosporins and fluoroquinolones, which would have exerted significant selection pressure for persistence of chromosomal fluoroquinolone mutations and presence of *bla*_CTX-M_. Sporadic gain/loss events involving other, non-FII *bla*_CTX-M-15_ plasmids have also occurred but have not dominated. Similar processes may be driving the more recent emergence of sublineages of ST131 with *bla*_CTX-M-14_ and *bla*_CTX-M-27_, as described in Japan (36), although for *bla*_CTX-M-14_, these appear to have occurred on at least two occasions (clades A and C1/*H*30R [Fig. 1]). This study highlights the global imperative to reduce antimicrobial selection pressures; the capacity of these resistance plasmids for genetic reassortment; the important role of certain insertion sequences, such as IS*26*, in facilitating horizontal mobility of resistance determinants; and the possibility of targeting specific replicons in an attempt to limit the spread of important resistance gene mechanisms.

## MATERIALS AND METHODS

### Sample collection, sequencing, and sequence read processing.

Isolates were obtained from wider collections held in several centers: the Shoklo Malaria Research Unit, Mae Sot, Thailand; the Lao-Oxford-Mahosot Hospital Wellcome Trust Research Unit, Vientiane, People’s Democratic Republic of Laos; the Cambodia-Oxford Medical Research Unit, Angkor Hospital for Children, Siem Reap, Cambodia; and the Microbiology Laboratory, Oxford University Hospitals NHS Trust, Oxford, United Kingdom. No two isolates were taken from the same individual. In addition, seven isolates collected from clinical samples across Canada between 2006 and 2008 and one isolate recovered from poultry in 2006 were included. DNA was extracted as previously described (38). Sequence data for the eight AstraZeneca strains had been generated from a series of isolates collected by International Health Management Associates, Inc., as part of a global resistance survey; the data for the Price strains were as previously described (13). Sequencing was performed using either the Illumina HiSeq or the MiSeq sequencer (100- or 151-bp paired-end reads [details for non-Price strains are in Table S1 in the supplemental material]). Sequence type was confirmed using BLASTn-based (39) *in silico* multilocus sequence typing (MLST) of *de novo*-assembled WGS data (40).

Properly paired sequence reads were mapped using Stampy v1.0.17 (without Burrows-Wheeler Aligner premapping, using an expected substitution rate of 0.01) to a fully sequenced *E. coli* ST131 reference (*E. coli* O150:H5 SE15; RefSeq NC_013654), in order to limit bias introduced by mapping to a more divergent reference. Repetitive regions (166,828 bases, 3.5%) of the reference were identified using self-self BLASTn analysis with default settings; these regions were then masked prior to mapping and base calling. Single-nucleotide variants (SNVs) were determined across all mapped nonrepetitive sites using SAMtools (version 0.1.18) mpileup. mpileup was run twice to separate high-quality base calls from low-quality base calls: first, with options “-E -M0 -Q25 -q30 -m2 -D -S” and otherwise default settings, and second, with options “-B -M0 -Q0 -q0 -m2 -D -S” and otherwise default settings. Vcf files of annotated variant sites were created using GATK (v1.4.21). Base calls derived from these two Vcf files were then retained only if (i) the proportion of high-quality bases supporting the call was ≥90%, and ≥5 high-quality bases were required as a minimum; (ii) the root of the mean square mapping quality of reads covering a putative variable site was ≥30; (iii) the Phred scaled quality supporting a base call was ≥25; and (iv) reads spanning the putative variable site were made up of ≥35% high-quality bases. Core variable sites (base called in all sequences, excluding “N” or “-” calls) derived from mapping to the SE15 reference were “padded” with invariant sites in a proportion consistent with the GC content and length of the reference genome (4.72 Mb, 51% average GC content), to generate a modified alignment of input sequences for our phylogenetic analyses (see below).

*De novo* assemblies were generated using Velvet with the VelvetOptimiser wrapper (*n* = 211) (41) (http://bioinformatics.net.au/software.velvetoptimiser.shtml) or A5-MiSeq (42). The latter was used in cases where the number of assembled bases was below the expected assembly size of 4 to 5.5 Mb (*n* = 4 [strains la_12107_3, can_70883, can_1731_01, and can_1070] in which the median optimized assembly size with Velvet was 16,004 bases and the median number of contigs was only six). Using A5-MiSeq, assemblies for these four strains were generated with an appropriate median size of 5,143,908 bp and 269 contigs.

### Identification/characterization of *bla*_CTX-M_ and genetic context, *gyrA* mutations, and *fimH* typing.

BLASTn analysis of *de novo* assemblies was used to identify: (i) *bla*_CTX-M_ presence and variants (in-house reference gene database) (38); (ii) genetic context for *bla*_CTX-M_, by extracting and annotating contigs containing *bla*_CTX-M_ variants using PROKKA and ISFinder (manual annotation) (43, 44); (iii) chromosomal *gyrA* mutations in the quinolone resistance-determining region known to be responsible for conferring most resistance to fluoroquinolones; (iv) *fimH* presence and variant (45); and (v) Inc type using the downloaded PlasmidFinder (46) and pMLST databases (available at http://pubmlst.org/plasmid/) (47). Genetic contexts for *bla*_CTX-M_ were classified as chromosomal if annotations for regions flanking *bla*_CTX-M_ were found to be consistently chromosomal in other *E. coli* strains in GenBank and plasmid if these were associated specifically with plasmids (e.g., *tra* genes); otherwise, they were classified as unknown. IncFII_AY458016-like sequences were extracted, aligned, and visually inspected to confirm variant types using Geneious (version 7.1.9; Biomatters Ltd., Auckland, New Zealand) (48).

### ST131 chromosomal phylogenetic comparisons using ClonalFrame, BEAST, and BASTA.

ExPEC bacteria are recombinogenic and contain recombination hot spots with higher-than-average recombination rates (49). Recombination can obscure the clonal phylogenetic signal, and we therefore initially analyzed the alignment of sequences with ClonalFrame (50) to identify recombinant regions. Three separate runs were performed on the alignment of ST131 sequences, with the following settings: 2,000 burn-in iterations, 2,000 Monte Carlo Markov chain (MCMC) iterations following the burn-in period, and 2 iterations between recording parameter values in the posterior sample (the thinning interval). Convergence of the runs was assessed by comparing the similarities of the run outputs. SNVs within regions identified as recombinant from the consensus ClonalFrame output were ignored in the subsequent BEAST/BASTA analyses.

Using the modified alignment of ST131 sequences generated following the ClonalFrame analysis, mutation rate estimates across ST131 and a time-scaled phylogeny were calculated in BEAST (51). The model parameters were (i) a generalized time-reversible nucleotide substitution model; (ii) four relative rates of mutation across sites, allowing for all sites to be subject to mutation (i.e., the proportion of invariant sites fixed at 0%); (iii) a strict molecular clock estimating a uniform evolutionary rate across all branches of the tree; and (iv) a constant population size. Triplicate runs with 30 million iterations were performed, with 10% discounted as burn-in. Run convergence and mixing were assessed by inspecting the run log files in Tracer v1.5 (http://beast.bio.ed.ac.uk); adequate convergence of run statistics and mixing for each run and effective sample sizes (ESSs) for all parameters greater than 200 were required for an analysis to be considered adequate, in line with recommendations in the BEAST tutorials on the developers’ website (http://beast.bio.ed.ac.uk). We explored the application of several other models in BEAST incorporating the relaxed clock and variable population growth (exponential, logistic, and Bayesian skyride), but these either failed to converge, showed poor mixing, or had effective sample size (ESS) estimates of <200 and were therefore not considered robust.

We used the phylogeographic method BASTA (20) in the Bayesian phylogenetic package BEAST 2.2.1 (52) to infer patterns and rates of migration between geographical regions from the genome alignment, collection dates, and sampling locations. Initially, we grouped samples into three discrete locations, North America, Southeast Asia, and Europe, and disregarded samples from South America and Australasia because of the small sample numbers. Due to the nonrandom sampling scheme, we estimated only a single effective population size, equal for all locations, and a symmetric migration rate matrix. The analysis was run for 10^8^ MCMC steps. We subsequently reran the analysis including a fourth, unsampled deme, using the same model parameters, to determine whether this altered the outcome.

### Plasmid transformations, sequencing, and analyses.

Plasmid transformants were generated from 30 strains chosen on the basis of tree topology and association with CTX-M variants, aiming to transform at least one plasmid from each of the major CTX-M variant clusters. Two *bla*_CTX-M_-containing plasmids from non-ST131 *E. coli* (one ST617/*bla*_CTX-M-15_ and one ST405/*bla*_CTX-M-55_) were also transformed and sequenced as an external comparison.

Plasmid DNA was extracted from subcultures of frozen stock grown overnight on blood agar, followed by selective culture of a single colony in lysogeny broth (BD/Difco LB broth; Miller [Luria-Bertani]; catalog no. 244620) with ceftriaxone at 1 µg/ml. DNA extraction was performed using the Qiagen plasmid minikit (Qiagen, Venlo, Netherlands), in accordance with the manufacturer’s instructions, with the addition of Glycoblue coprecipitate (Life Technologies, Carlsbad, CA, USA) to the DNA eluates prior to isopropanol precipitation to enable better visualization of the DNA pellet. Plasmid DNA was redissolved in distilled water and then typically electroporated on the same day or stored in the refrigerator prior to electroporation within 24 h.

Commercially prepared DH10B *E. coli* (ElectroMAX DH10B cells; Invitrogen/Life Technologies, Carlsbad, CA) was used as the recipient cell strain for plasmid electroporation, because of its high transformation efficiency and the fact that the strain has been fully sequenced (NCBI RefSeq NC_010473.1) (53). Electroporation was performed with a MicroPulser electroporator (Ec2 settings). Transformant cell suspensions were cultured on selective agar (Luria-Bertani agar plus ceftriaxone [1 µg/ml]), with appropriate controls.

Sequencing was performed on the Illumina HiSeq or MiSeq sequencer, generating 150- or 300-base paired-end reads (see Table S2 in the supplemental material). Sequencing reads from the isolate from which the transformed plasmid had been obtained were mapped back to the transformed plasmid assembly in order to ascertain the reliability of the assembly in each case. Reads were assembled using A5/A5-MiSeq (42), and assembled contigs were annotated with PROKKA (43). The median plasmid assembly size was 122,786 (range, 72,449 to 171,919), with a median of 22 contigs in each assembly (range, 1 to 33). Using longer reads (300 bp; MiSeq platform) resulted in a significantly smaller number of contigs per assembly (median, 17 versus 25; rank sum *P* = 0.003). Mapping was used to assess the reliability of our plasmid constructs and reflected the content present in each transformed and assembled resistance plasmid, with the exception of 8A16G_T.

A single strain (P46212) from the data set was also sequenced using long-read technology (PacBio); the CTX-M-15 plasmid (pP46212) from this strain was assembled into a single, circularized contig as described elsewhere (A. E. Sheppard, N. Stoesser, D. J. Wilson, R. Sebra, A. Kasarskis, L. W. Anson, A. Giess, L. J. Pankhurst, A. Vaughan, C. J. Grim, H. L. Cox, A. J. Yeh, Modernising Medical Microbiology Informatics Group, C. D. Sifri, A. S. Walker, T. E. A. Peto, D. W. Crook, and A. J. Mathers, submitted for publication).

Plasmid content across the data set was investigated in a number of ways. First, the transformed plasmid sequences that we generated were used as references against which BLASTn-based comparisons for degree of presence/absence were made for the whole data set. We used default BLASTn settings to compare the *de novo* assembly for each ST131 isolate with each respective, concatenated plasmid reference sequence. Sequence identity of BLASTn hits across the plasmid reference was plotted using the heatmap.2 package in R, with a minimum threshold of 90% identity for plotting. For simplicity, values were averaged over 100-bp bins (script available at https://github.com/aesheppard/plasmid_comp).

Second, comparisons between each pair of transformed plasmid assemblies were undertaken, again using BLASTn with default settings. For the query sequence in each comparison, the percentage of sites contained in hits (counting overlapping hits only once) was identified from parsed blast output. For each pair, two percentage of homology statistics were generated, taking each member of the pair as a reference in turn, to account for differences in length (script available at https://github.com/aesheppard/plasmid_comp). The mean percent divergence for each plasmid sequence pair was then plotted against the time to most recent common ancestor (TMRCA) of the two host strains containing those transformed plasmid sequences (derived from the time-scaled tree) in Stata (SE) (StataCorp, Texas, USA; version 11.2).

Third, for visualization, plasmid sequences were compared using ProgressiveMauve (54), with assembled contigs reordered with respect to the pP46212 PacBio-generated CTX-M-15 plasmid reference, using the “Move contigs” tool. For this, three transformed plasmid sequences were excluded: 8A16G_T because of issues surrounding the assembly, 11B01979_T because it was virtually identical to transformed *bla*_CTX-M-14_ plasmid sequences in clade A, and 19B19L_T because it lacked an FII replicon. Finally, annotated, transformed plasmid sequences were clustered using CD-Hit (55) [-c 1.0 -n 5 -d 0 -g 1], to identify whether any coding sequences were shared and whether there might be any biological significance associated with these on the basis of their annotations.

### Sequencing data resources.

The positions for called, variable sites across the data set (with respect to the reference SE15 *E. coli* genome) are listed in Text S1 in the supplemental material, and the positions in recombinant regions (and therefore not included in the phylogenetic analyses) are listed in Text S2. Contigs for the *de novo* assemblies for the transformed plasmid sequences are in Text S3, and those for all the new isolates are freely downloadable at http://modmedmicro.nsms.ox.ac.uk/stoesser-n-et-al/.

### Accession numbers.

Sequencing data for the new isolates sequenced for this study have been deposited in the NCBI Short Read Archive (BioProject number PRJNA297860, 108 ST131 sequences and 30 *bla*_CTX-M_ plasmid transformants [see Tables S1 and S2 in the supplemental material]). The uk_P46212 sequence assembled using PacBio is available from GenBank (accession numbers CP013658 [chromosome] and CP013657 [CTX-M-15 plasmid]).

## SUPPLEMENTAL MATERIAL

Figure S1  Genetic contexts of clade A-associated *bla*_CTX-M-14/14-like_ variants. Many contexts are limited by the extent of the assembled region around the *bla*_CTX-M-14/14-like_ gene (marked with “X”). For all aligned, similarly colored regions, sequence homology is preserved; curly brackets cluster those isolates with identical flanking sequences. Flanking contexts not shown for isolates with known chromosomal integration or for *bla*_CTX-M_-negative/non-*bla*_CTX-M-14/14-like_ isolates in the subclusters. Coloring of isolate names reflects geographic locations (blue, North America; red, Europe; green, Southeast Asia; yellow, Australasia). Download Figure S1, PDF file, 0.2 MB

Figure S2  Genetic contexts of clade C1-associated *bla*_CTX-M-14/14-like_ variants. Many contexts are limited by the extent of the assembled region around the *bla*_CTX-M-15_ gene (marked with “X”). For all aligned, similarly colored regions, sequence homology is preserved; curly brackets cluster those isolates with identical flanking sequences. Flanking contexts not shown for isolates with known chromosomal integration or for *bla*_CTX-M_-negative/non-*bla*_CTX-M-14/14-like_ isolates in the subcluster (blue, North America; red, Europe; green, Southeast Asia; yellow, Australasia). Download Figure S2, PDF file, 0.2 MB

Figure S3  Inc types identified in whole-isolate sequencing data, plotted with respect to ST131 host strain phylogeny. Blast match (%) denotes a composite score of percent matched length and percent homology to reference Inc sequence, with highest percent score/contig hit represented. Matches of <80% were excluded. Reference Inc sequences were downloaded from the PlasmidFinder database; those that were present (Blast match of ≥80%) in at least one isolate are represented on the *x* axis. Download Figure S3, PDF file, 0.5 MB

Figure S4  BLASTn-based comparisons across the ST131 data set, using la_12107-3_T and la_5220-3_T as references. Color represents degree of presence/absence of corresponding reference sequence on an isolate-by-isolate basis per row. Rows/isolates arranged as in the Fig. 1 phylogeny. Download Figure S4, PDF file, 2.3 MB

Text S1  List of positions for called, variable sites across the data set, with respect to the reference SE15 *E. coli* genome. Download Text S1, TXT file, 0.05 MB

Text S2  List of positions identified as being in recombinant regions through the ClonalFrame analysis. Download Text S2, TXT file, 4.5 MB

Text S3  Fasta file of contigs for the *de novo* assemblies for the transformed plasmid sequences. Download Text S3, TXT file, 3.4 MB

Table S1  Details of newly sequenced ST131 strains included in the analysis. Table S1, XLSX file, 0.1 MB

Table S2  Details of transformed *bla*_CTX-M_ plasmid sequences. Table S2, XLSX file, 0.04 MB
