# Supplementary figures and images for: Evolutionary History of the Global Emergence of the Escherichia coli Epidemic Clone ST131
Source: mBio. 2016 Mar 22;7(2):e02162-15. doi: 10.1128/mBio.02162-15 (PMC4807372; doi:10.1128/mBio.02162-15)

### CLADE A SUB-CLUSTER [i], CTX-M-14

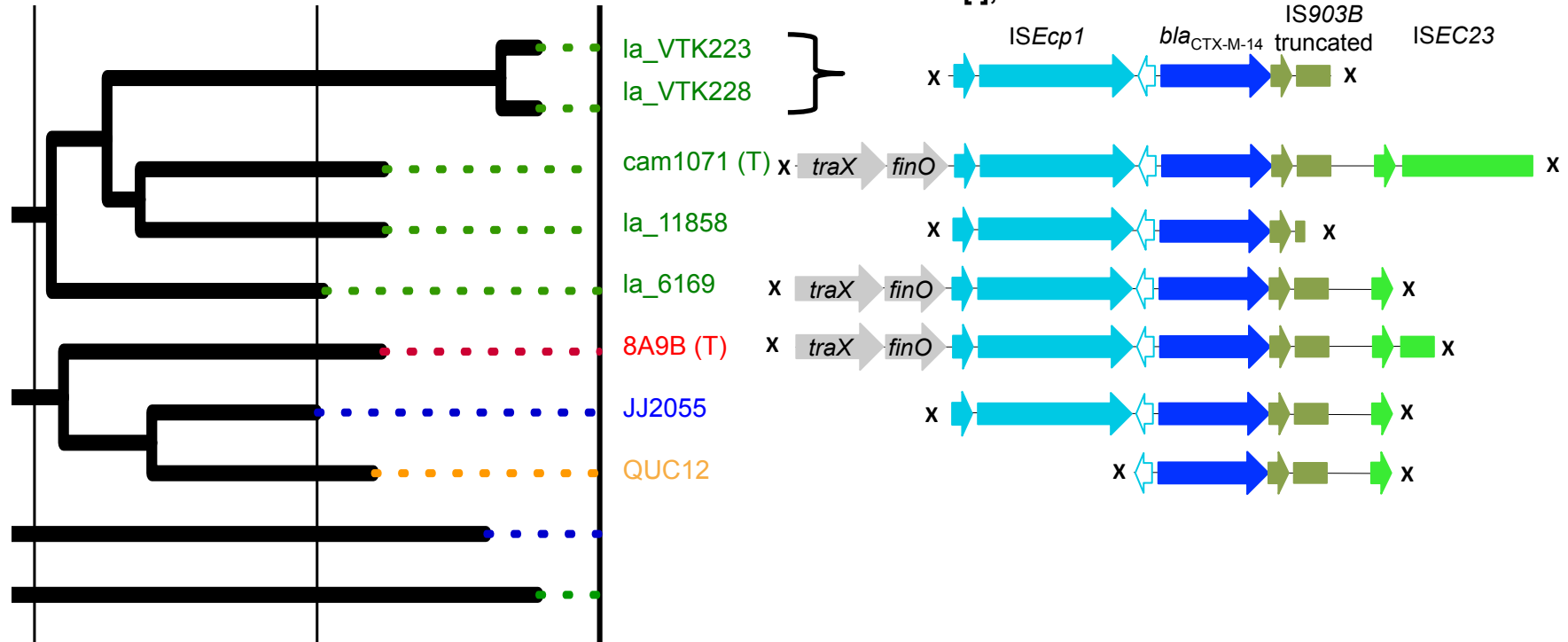

### CLADE A SUB-CLUSTER [ii], CTX-M-27

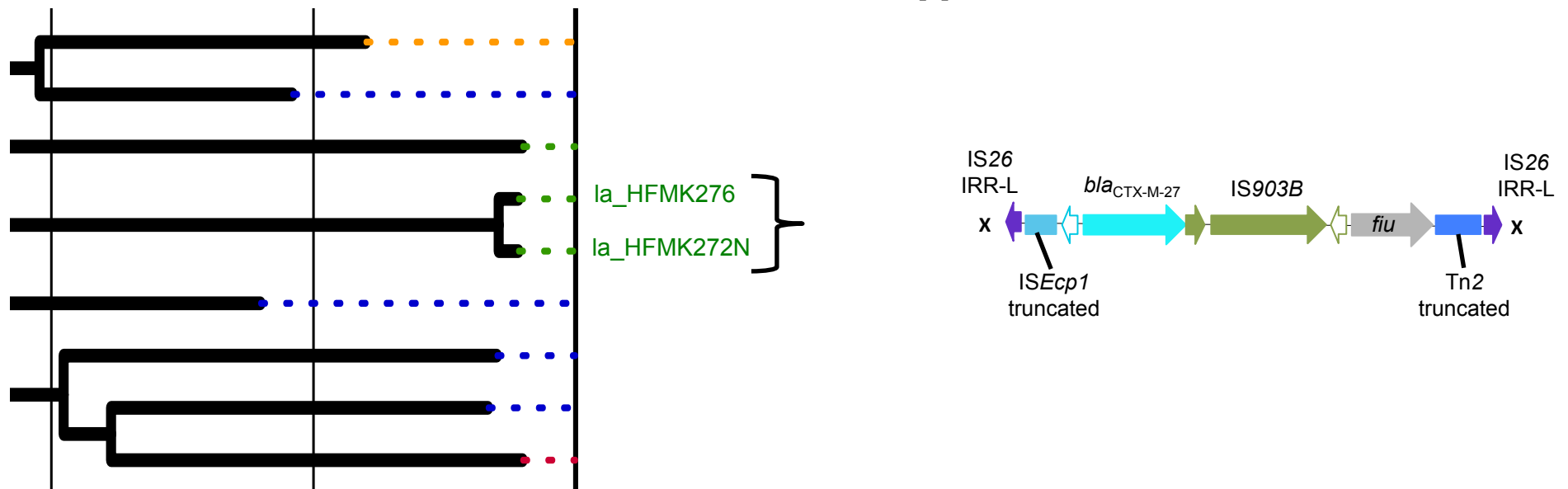

Supplement: Figure S1 — Genetic contexts of clade A-associated blaCTX-M-14/14-like variants. Many contexts are limited by the extent of the assembled region around the blaCTX-M-14/14-like gene (marked with “X”). For all aligned, similarly colored regions, sequence homology is preserved; curly brackets cluster those isolates with identical flanking sequences. Flanking contexts not shown for isolates with known chromosomal integration or for blaCTX-M-negative/non-blaCTX-M-14/14-like isolates in the subclusters. Coloring of isolate names reflects geographic locations (blue, North America; red, Europe; green, Southeast Asia; yellow, Australasia). Download [file mbo002162744sf1.pdf]

# CLADE C1 CTX-M-14 and CTX-M-27 SUB-CLUSTERS

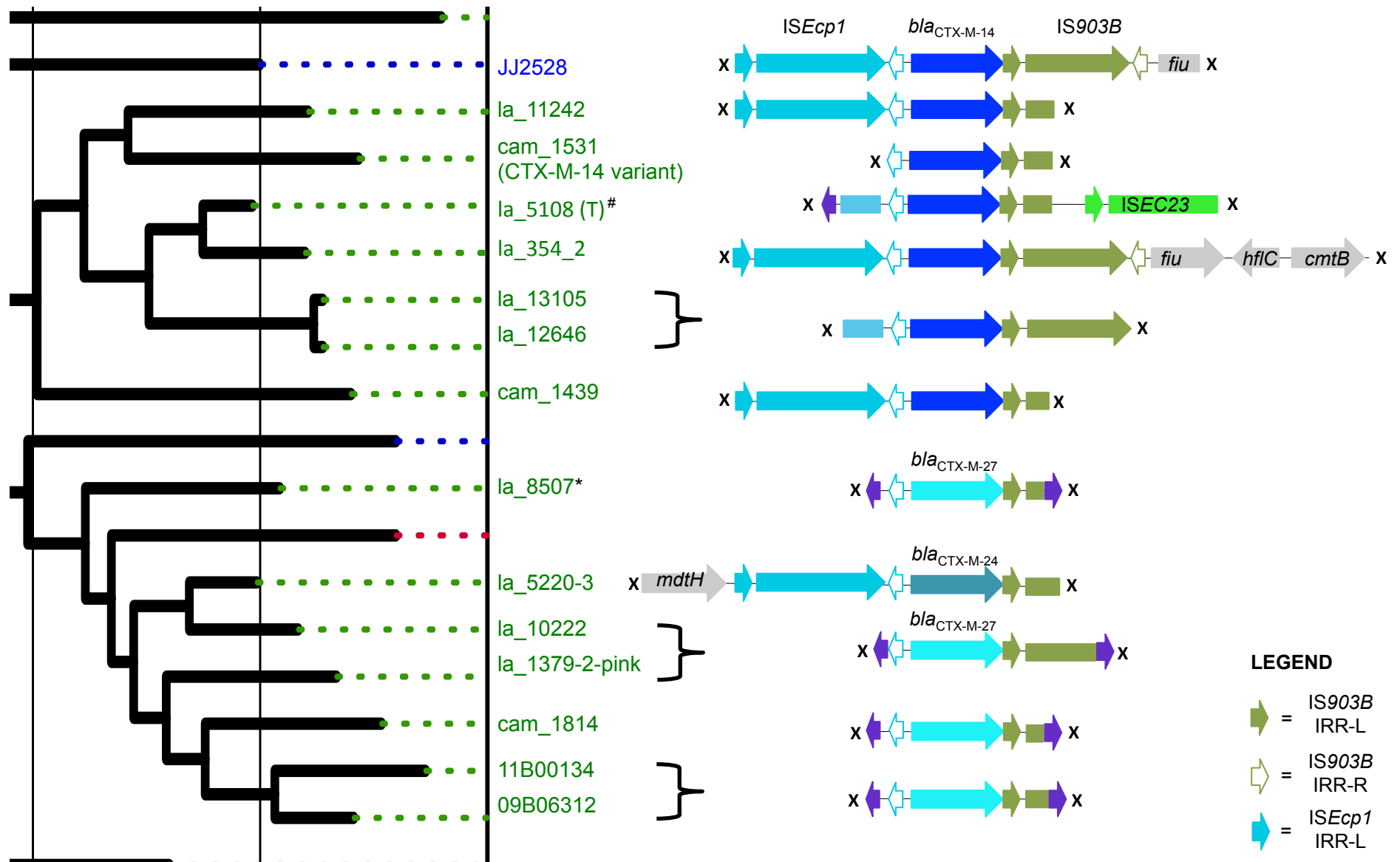

\* Same as 11B00134 and 09B03612

# Similar to clade A CTX-M-14 sub-cluster [i]

Supplement: Figure S2 — Genetic contexts of clade C1-associated blaCTX-M-14/14-like variants. Many contexts are limited by the extent of the assembled region around the blaCTX-M-15 gene (marked with “X”). For all aligned, similarly colored regions, sequence homology is preserved; curly brackets cluster those isolates with identical flanking sequences. Flanking contexts not shown for isolates with known chromosomal integration or for blaCTX-M-negative/non-blaCTX-M-14/14-like isolates in the subcluster (blue, North America; red, Europe; green, Southeast Asia; yellow, Australasia). Download [file mbo002162744sf2.pdf]
