# Supplementary material for: Evolutionary History of the Global Emergence of the Escherichia coli Epidemic Clone ST131
Source: mBio. 2016 Mar 22;7(2):e02162-15. doi: 10.1128/mBio.02162-15 (PMC4807372; doi:10.1128/mBio.02162-15)
Supplement: Figure S3 — Inc types identified in whole-isolate sequencing data, plotted with respect to ST131 host strain phylogeny. Blast match (%) denotes a composite score of percent matched length and percent homology to reference Inc sequence, with highest percent score/contig hit represented. Matches of <80% were excluded. Reference Inc sequences were downloaded from the PlasmidFinder database; those that were present (Blast match of ≥80%) in at least one isolate are represented on the x axis. Download [file mbo002162744sf3.pdf]

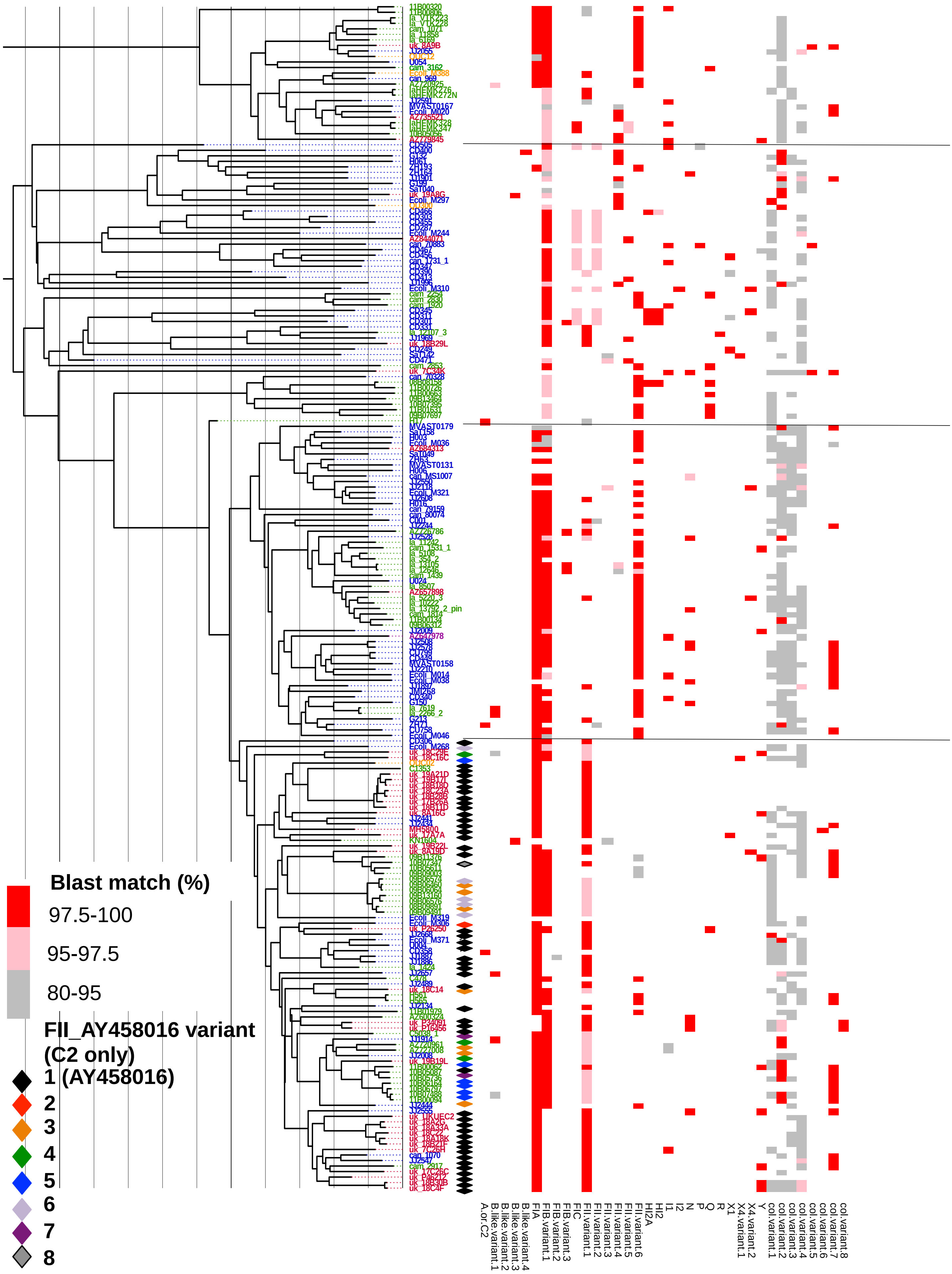

Blast match (%)
97.5-100
95-97.5
80-95
FII_AY458016 variant
(C2 only)
1 (AY458016)
2
3
4
5
6
7
8
A.or.C2
B.like.variant.1
B.like.variant.2
B.like.variant.3
B.like.variant.4
FIA
FIB.variant.1
FIB.variant.2
FIB.variant.3
FIC
FII.variant.1
FII.variant.2
FII.variant.3
FII.variant.4
FII.variant.5
FII.variant.6
HI2
HI2A
I1
I2
N
P
Q
R
X1
X4.variant.1
X4.variant.2
Y
col.variant.1
col.variant.2
col.variant.3
col.variant.4
col.variant.6
col.variant.7
col.variant.8

PlasmidFinder sequence	Listing in Figure S4
A/C2_1__JN157804	A or C2
B/O/K/Z_1__CU928147	B-like variant 1
B/O/K/Z_3_GQ259888	B-like variant 2
B/O/K/Z_2_GU256641	B-like variant 3
B/O/K/Z_4_FN868832	B-like variant 4
FIA_1__AP001918	FIA
FIB(AP001918)_1__AP001918	FIB variant 1
FIB(pB171)_1_pB171_AB024946	FIB variant 2
FIB(pLF82)_1_pLF82_CU638872	FIB variant 3
FIC_1__AP001918	FIC
FII_1__AY458016	FII variant 1
FII(pSE11)_1_pSE11_AP009242	FII variant 2
FII(pCoo)_1_pCoo_CR942285	FII variant 3
FII(29)_1_pUTI89_CP003035	FII variant 4
FII(pHN7A8)_1_pHN7A8_JN232517	FII variant 5
FII(pRSB107)_1_pRSB107_AJ851089	FII variant 6
HI2A_1__BX664015	HI2A
HI2_1__BX664015	HI2
I1_1_Alpha_AP005147	I1
I2_1_Delta_AP002527	I2
N_1__AY046276	N
P_1_alpha_L27758	P
Q1_1__HE654726	Q
R_1__DQ449578	R
X1_1__EU370913	X1
X4_1__CP002895	X4 variant 1
X4_2__FN543504	X4 variant 2
Y_1__K02380	Y
ColRNAI_1__DQ298019	col variant 1
Col156_1__NC_009781	col variant 2
Col8282_1__DQ995353	col variant 3
Col(MG828)_1__NC_008486	col variant 4
ColpVC_1__JX133088	col variant 5
Col(IMGS31)_1__NC_011406	col variant 6
Col(BS512)_1__NC_010656	col variant 7
Col(KPHS6)_1__NC_016841	col variant 8
