# Supplementary material for: Evolutionary History of the Global Emergence of the Escherichia coli Epidemic Clone ST131
Source: mBio. 2016 Mar 22;7(2):e02162-15. doi: 10.1128/mBio.02162-15 (PMC4807372; doi:10.1128/mBio.02162-15)
Supplement: Figure S4 — BLASTn-based comparisons across the ST131 data set, using la_12107-3_T and la_5220-3_T as references. Color represents degree of presence/absence of corresponding reference sequence on an isolate-by-isolate basis per row. Rows/isolates arranged as in the Fig. 1 phylogeny. Download [file mbo002162744sf4.pdf]

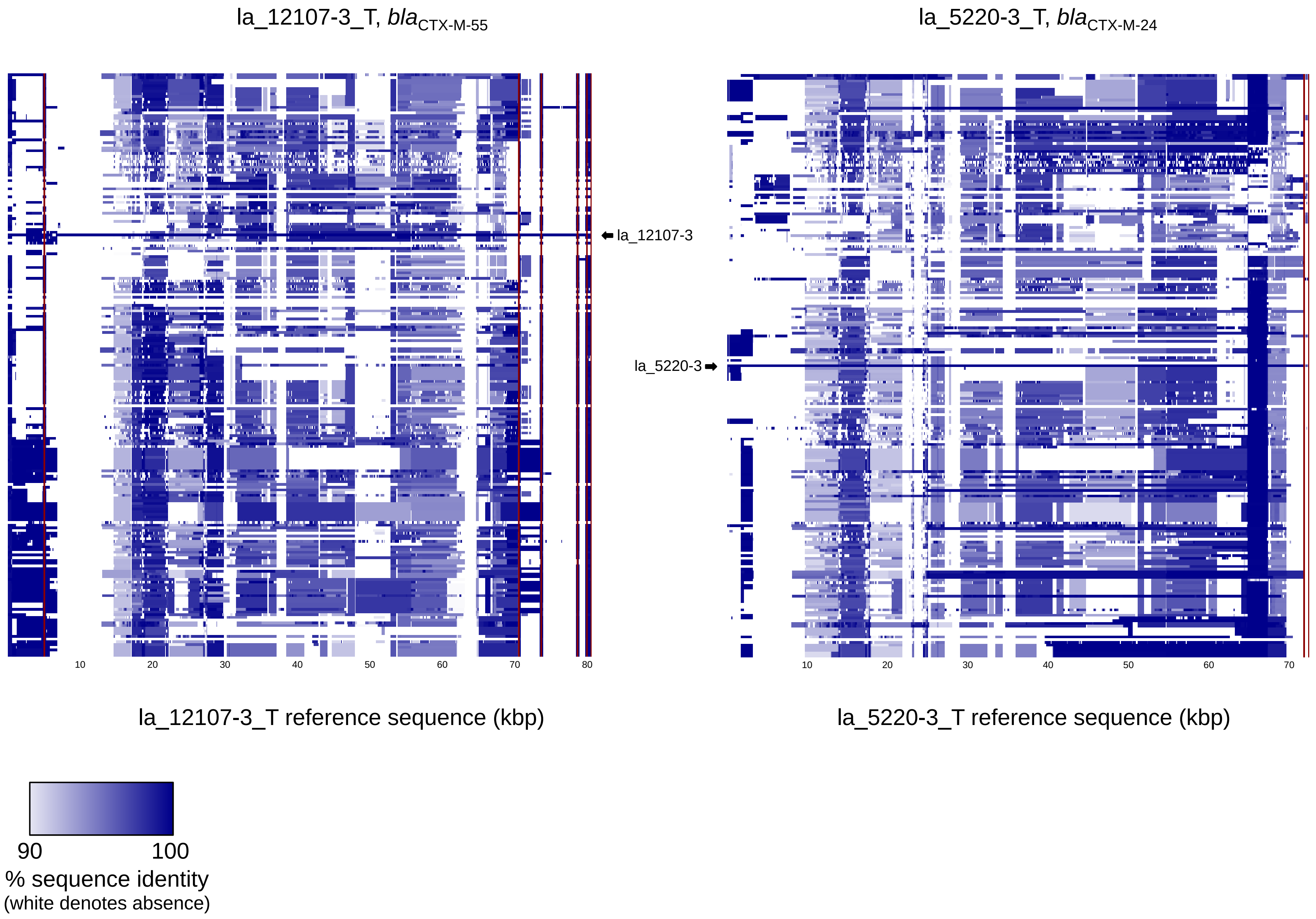
la_12107-3_T, *bla*CTX-M-55
la_5220-3_T, *bla*CTX-M-24
la_12107-3
la_5220-3
10
20
30
40
50
60
70
80
10
20
30
40
50
60
70
la_12107-3_T reference sequence (kbp)
la_5220-3_T reference sequence (kbp)
90
100
% sequence identity
(white denotes absence)
